# Attacked from All Sides: RNA Decay in Antiviral Defense

**DOI:** 10.3390/v9010002

**Published:** 2017-01-04

**Authors:** Jerome M. Molleston, Sara Cherry

**Affiliations:** Department of Microbiology, University of Pennsylvania Perelman School of Medicine, Philadelphia, PA 19104, USA; jeromem@mail.med.upenn.edu

**Keywords:** RNA decay, RNA-protein interactions, decapping, Xrn1, exosome, TRAMP, exonuclease, RNAse, intrinsic immunity, antiviral

## Abstract

The innate immune system has evolved a number of sensors that recognize viral RNA (vRNA) to restrict infection, yet the full spectrum of host-encoded RNA binding proteins that target these foreign RNAs is still unknown. The RNA decay machinery, which uses exonucleases to degrade aberrant RNAs largely from the 5′ or 3′ end, is increasingly recognized as playing an important role in antiviral defense. The 5′ degradation pathway can directly target viral messenger RNA (mRNA) for degradation, as well as indirectly attenuate replication by limiting specific pools of endogenous RNAs. The 3′ degradation machinery (RNA exosome) is emerging as a downstream effector of a diverse array of vRNA sensors. This review discusses our current understanding of the roles of the RNA decay machinery in controlling viral infection.

## 1. Viral RNAs (vRNAs) as Foreign RNAs

RNA viruses produce RNAs which differ substantially from normal cellular RNAs, leading to their recognition by host-encoded RNA-binding proteins. Unlike cellular RNAs, the genomes of RNA viruses are replicated by RNA-dependent RNA polymerases (RdRp) through antigenome intermediates, creating both transient double-stranded RNA (dsRNA) structures and 5′ triphosphate ends not normally present in cellular messenger RNAs (mRNAs) [1]. For 5′ end protection and recruitment of the translational machinery, endogenous mRNAs are capped in the nucleus. However, cytoplasmic RNA viruses have no access to the normal cellular capping machinery and, thus, many of these viruses go to great lengths to acquire a cap, including enzymatically synthesizing a cap or cap mimic, or acquiring one from cellular mRNA through a process known as cap-snatching [2]. For translation and 3′ end protection, most mRNAs are polyadenylated; some cytoplasmic viral mRNAs achieve this through RdRp-mediated polyadenylation [3]. Furthermore, some viruses encode 3′ structures which impede exonucleases [4,5]. These are but a few examples of the complexity of viral RNA (vRNA) metabolism, which can render viruses susceptible to both immune sensors and the cellular RNA decay machinery.

## 2. Innate Immune Recognition of vRNA

### 2.1. RIG-I Like Receptors (RLRs) and DEAD-Box Helicases

The RNAs produced during viral replication serve as an important sign of infection, and a series of sensors have evolved to detect these RNAs. In mammals, many cytosolic vRNAs are recognized by the RIG-I-like receptors (RLRs), RIG-I and MDA-5, which are homologous DEAD-box RNA helicases [6]. Each recognizes different RNA structures. RIG-I recognizes short dsRNAs and RNAs with 5′ triphosphates, and plays a role in restricting viruses, including paramyxoviruses, orthomyxoviruses, and flaviviruses [7,8]. In contrast, MDA-5 recognizes longer dsRNAs and higher-order RNA structures, and is integral for recognition of picornaviruses [9,10]. Both can respond to the synthetic dsRNA polyinosinic-polycytidylic acid (poly(I:C)) dependent on length; long poly(I:C) is a ligand of MDA-5, while short poly(I:C) can activate RIG-I [11]. The RLRs primarily act by signaling the interferon (IFN) system through their adaptor, MAVS; IFN then signals an extensive transcriptional antiviral program [12,13,14,15]. A third RLR helicase, LGP2, lacks the ability to signal through MAVS, and in different contexts has been found to either inhibit or potentiate the signaling of the other two RLRs [16,17,18,19,20,21,22]. In response, many RNA viruses have evolved mechanisms to evade the IFN system and, thus, avoid the consequences of RLR detection; for example, both Rift Valley fever virus (RVFV) and Sindbis virus (SINV) encode accessory proteins (NSs and nsP2) which inhibit the transcription of IFNs [23,24,25,26,27].

Just as mammalian cells utilize the RLR DEAD-box RNA helicases to recognize cytosolic vRNA, the closest *Drosophila* homolog, Dicer-2, recognizes viral dsRNA intermediates generated during infection [28,29,30]. However, Dicer-2 functions as both sensor and effector; in addition to its helicase domain, it has a ribonuclease (RNase) III domain which cleaves dsRNAs into siRNAs which, in turn, are loaded into the Argonaute 2-containing RNA-induced silencing complex, preventing RNA translation and cleaving vRNA [31]. The antiviral RNA silencing pathway in *Drosophila* is essential for immune defense; flies with mutations in this pathway rapidly succumb to viral infection. To evade this immune defense, natural insect pathogens such as *Drosophila* C virus encode suppressors of RNAi [29,32]. Dicer-2 also has silencing-independent antiviral functions which closely parallel the signaling functions of the RLRs; Dicer-2 is required to induce the transcription of the antiviral factor Vago, suggesting that it is also a regulator of antiviral transcription during viral infection in insects [33].

New roles are continually emerging for the larger family of DEAD-box helicases in recognizing vRNA. Many of these genes have roles both in normal cellular metabolism as well as in the control of viral infection. For example, DDX17 normally binds stem-loop structures of primary microRNAs (pri-miRNAs) in the nucleus and recruits the Drosha-anchored microprocessor complex to produce pre-miRNAs [34,35,36,37]. However, upon viral infection, DDX17 is repurposed and exported to the cytoplasm, where it binds a stem-loop miRNA-like structure in RVFV RNA in order to restrict viral replication [37]. Additional helicases are involved in innate recognition, such as the DEAD-box helicase DDX60, which interacts with RIG-I, and the complex of DDX1, DDX21, and DHX36, which bind the innate immune adaptor TRIF, in each case facilitating their activity [38,39].

### 2.2. Toll-Like Receptors (TLRs)

Endosomal RNAs are sensed by toll-like receptors (TLRs) including TLR3 and TLR7, which detect dsRNA and ssRNA, respectively. These sensors signal through the adaptors TRIF and MyD88 to activate antiviral transcription programs [6]. Endosomal TLRs are highly expressed in dedicated immune cells such as dendritic cells, but are missing from many other cell types, which can only effectively sense cytosolic RNA [40,41,42].

### 2.3. Protein Kinase R (PKR)

Mammalian cells possess additional cytosolic sensors of vRNA including the dsRNA-activated protein kinase R (PKR) [43,44]. Activation of PKR by dsRNAs from viruses or poly(I:C) induces autophosphorylation of PKR and subsequent phosphorylation of eIF2α, shutting down global protein translation thereby preventing viral protein synthesis [45,46,47,48]. Many viruses prevent PKR-mediated translational shutdown by binding dsRNA or PKR to prevent its activation [44,49]. Other viruses, such as RVFV and poliovirus, induce PKR degradation [50,51,52]. In contrast, several other viruses, such as hepatitis C virus (HCV) and SINV, encode RNA structures which bypass the PKR-dependent global translational arrest and continue to be efficiently translated under these stress conditions [27,53,54,55,56]. Moreover, studies have found that RLR-dependent transcriptional activation is dependent on PKR and vice-versa, suggesting crosstalk between these pathways [57,58].

### 2.4. Ribonucleases (RNases)

Another cytoplasmic sensor of viral dsRNA, 2′-5′ oligoadenylate synthetase (OAS), is an IFN-inducible enzyme and, thus, is up-regulated in response to viral detection by sensors, such as the RLRs and TLRs [59,60]. Upon sensing vRNA, OAS synthesizes 2,5-adenylate, which, in turn, activates the latent cytoplasmic endoribonuclease RNASEL. RNASEL cleaves vRNA and cellular RNA, thereby inhibiting viral replication [61,62,63]. Furthermore, these cleaved RNAs can, in turn, act as substrates for RLR detection, amplifying the antiviral program [64]. In addition, RNASEL promotes apoptosis in response to viral infection, preventing further viral spread [65]. Recent studies have begun to characterize RNAs as more or less susceptible to RNASEL and to postulate further functions for RNASEL-mediated regulation of specific RNAs [66].

Drosha, a nuclear RNase III enzyme, has a canonical role in processing pri-miRNAs to pre-miRNAs before they are exported to the cytoplasm for Dicer processing. Recent studies have shown that Drosha has antiviral activity [67]. Drosha is exported to the cytoplasm in response to diverse RNA viruses, and restricts RNA virus infection by unknown mechanisms, although it is likely that Drosha is recognizing stem-loop structures in vRNA.

New research continues to uncover RNase activity among previously identified antiviral proteins. SAMHD1 was identified as a restriction factor for human immunodeficiency virus (HIV) that is antagonized by the viral protein Vpx [68]. An initial search for the antiviral mechanism revealed that SAMHD1 is a deoxynucleotide triphosphohydrolase that degrades DNA nucleotides, restricting the nucleotide pool available to HIV [69,70]. However, recent work has also identified 3′-5′ DNase and RNase activity for SAMHD1, suggesting additional antiviral functions that may be active against HIV and other viruses [71].

## 3. The Canonical RNA Decay Machinery and vRNA Targeting

Emerging data suggest that the canonical RNA decay machinery, which is largely dependent on exonucleases, also plays an important role in antiviral immunity. In general, RNA decay proceeds from either the 5′ or 3′ end of an RNA transcript, and has roles in RNA biogenesis, RNA quality control, and normal RNA turnover. Ribosomal RNAs (rRNAs), small nuclear RNAs (snRNAs), and small nucleolar RNAs (snoRNAs) all require RNA processing in the nucleus to reach their mature forms [72]. Quality control also begins in the nucleus, where RNAs which fail to be properly matured, such as hypoadenylated mRNAs and hypomodified tRNAs, are degraded before they can leave the nucleus [73,74]. In the cytoplasm, additional quality-control checkpoints, such as nonsense-mediated decay or no-go decay, detect stalled ribosomes or premature stop codons and degrade these aberrant mRNAs to release and recycle the translational machinery [75]. Recent studies have shown that RNA decay machinery also serves a key role in post-transcriptional regulation of groups of RNAs, called regulons, which are rapidly co-regulated through specific recognition of sequences in their 5′ and 3′ untranslated regions (UTRs) [76]. These include sequences such as terminal oligopyrimidines (TOPs) at the 5′ and AU-rich elements (AREs) at the 3′ ends of RNAs [77,78,79]. As viruses possess many features of aberrant RNAs, they are increasingly recognized as targets of the RNA decay machinery.

## 4. Antiviral Roles for Nonsense-Mediated Decay (NMD)

Nonsense-mediated decay (NMD) is the process by which mRNAs with stop codons far from the 3′ end of an mRNA, either due to a premature stop codon or a long 3′ UTR, are detected and degraded at either the 5′ or 3′ ends [75,80,81]. Several RNA viruses have been shown to be sensitive to this pathway. In particular, the NMD components Upf1, Smg5, and Smg7 restrict the replication of Semliki Forest virus (SFV) in mammalian cells [82]. The mechanism of this restriction is unclear, and may act through degradation of vRNA or indirect effects; it is independent of viral 3′ UTR length, suggesting that the long 3′ UTR of SFV mRNA is not necessary for NMD sensitivity. The antiviral effect of NMD is ancient; several plant NMD orthologs, including Upf1, were found to restrict potato virus X by recognizing vRNAs with long 3′ UTRs [83]. NMD is also antagonized by viruses; both HCV and human T-cell leukemia virus type 1 produce proteins which inhibit NMD, suggesting evolutionary pressure to evade this antiviral mechanism [84,85].

## 5. 5′ Decapping and Decay

Endogenous mRNAs targeted for 5′ decay are typically first deadenylated by the CCR4-NOT complex, often assisted by other deadenylating enzymes, before they can be targeted for decay [80,86]. Although deadenylation is the first regulated step towards mRNA degradation, it is sometimes reversible, and can act to “pause” mRNA translation rather than degrading these targets [87]. Removal of the 5′ cap of RNA by decappers (e.g., Dcp2) is irreversible, and permits degradation by the 5′–3′ exonucleases Xrn1 and Xrn2, in the cytoplasm and nucleus, respectively [88,89,90]. This process is largely conserved from yeast to mammals, though mammals have evolved multiple, partially-redundant decapping enzymes with specificity for different targets; Dcp2 is preferentially utilized in NMD and Nudt16 is preferentially involved in degradation of mRNAs containing AREs or 5′ TOPs [77,91].

Deadenylation, decapping, and 5′ degradation activities coalesce in ribonucleoprotein (RNP) structures known as processing bodies (P bodies). These RNPs consist of mRNAs targeted for decay as well as components of the decapping and 5′ degradation machinery (including Dcp2, its activators, and Xrn1) [86,92]. RNAs that accumulate in P bodies are removed from translation, and normally degraded [93]. Although P-bodies are present in normal cells at baseline, their number and size increase in response to a variety of stressors [94,95]. P bodies can interact with and exchange RNAs with other RNP granules, such as stress granules, which are composed of translationally-stalled RNAs and chaperone proteins [95,96]. There is evidence that visible P bodies are a consequence of high concentrations of mRNAs undergoing decay rather than being necessary for decay, as P body structure can be disrupted without preventing RNA degradation [97,98]. Furthermore, up-regulation of 5′ decapping and decay leads to the loss of visible P bodies due to the depletion of RNA targets [77]. These data suggest that P bodies are dynamic structures which form and dissolve in response to RNA target levels.

## 6. Antiviral Roles for 5′ to 3′ RNA Decay

**Figure 1:**
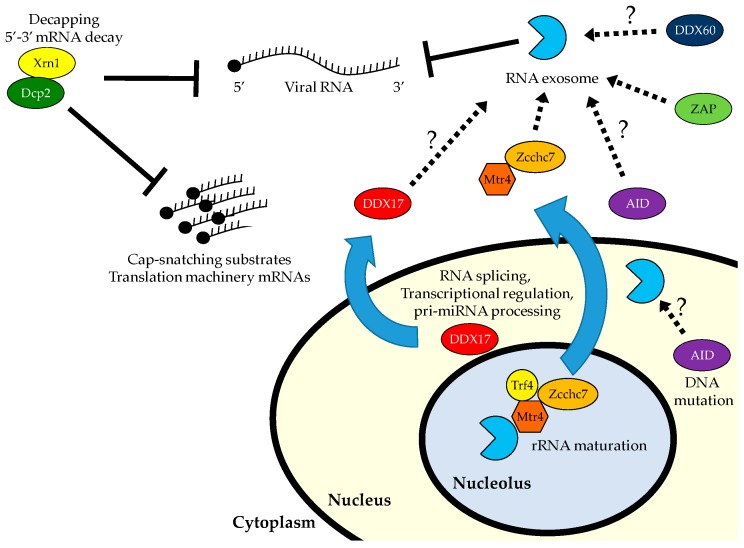
The 5′ to 3′ decay machinery can inhibit viral infection directly through degradation of viral RNA (vRNA; flaviviruses) or indirectly through decapping and degradation of RNAs needed for viral transcription and translation (bunyaviruses). The 3′ to 5′ decay machinery, the RNA exosome, interacts with a variety of RNA-binding proteins, some of which are exported to the cytoplasm in response to viral infection. Recruitment of the exosome can result in degradation of vRNA.

The 5′ to 3′ RNA decay machinery can inhibit viral replication in a number of different ways (Figure 1). Studies have shown that the cytoplasmic 5′ RNA exonuclease, Xrn1, can target flavivirus RNAs and in response these viruses antagonize Xrn1 by encoding structured RNAs that result in Xrn1 stalling [99,100,101]. Furthermore, poliovirus induces the degradation of host 5′ decay factors, such as Xrn1 and Dcp2, through a combination of viral and host proteases, suggesting evolutionary pressure to evade host 5′ RNA decay machinery [102]. In addition to directly targeting vRNAs, the 5′ decay machinery can also impact viral replication indirectly. Recent studies have shown that decappers limit the pool of host mRNAs available for RVFV to cap snatch from, attenuating replication in both insects and mammals [77,103]. Additionally, in mammals, RVFV infection induces NUDT16-mediated decapping and decay of 5′ TOP-containing mRNAs encoding the translational machinery, limiting both global and virus-specific translation [77].

P body structure is also altered during many viral infections. The up-regulation of 5′ decay during RVFV infection prevents the formation of P bodies due to depletion of the RNA targets around which they nucleate [77]. Poliovirus induces the degradation of 5′ decay proteins such as Xrn1 and Dcp1a, preventing P body formation [102]. In addition to the destruction of P bodies during some viral infections, P body components can be repurposed by viruses to facilitate infection. Flaviviruses relocalize P body components to viral replication centers, where they bind viral 3′ UTRs, promoting efficient viral replication [104,105,106].

## 7. The RNA Exosome

3′ to 5′ degradation is largely mediated by the RNA exosome. This complex consists of a hexameric barrel (six proteins with RNase PH homology) and a cap structure (three proteins with S1 RNA-binding domains) [107,108,109]. These structural components of the exosome form an internal channel wide enough to permit entry of single-stranded but not double-stranded RNA [110]. In contrast to the 5′ decay machinery which localizes to cytoplasmic P bodies, exosome components are both nuclear and cytoplasmic, and can accumulate in poorly-understood RNP granules [92,111,112,113]. While exosome proteins share structural and sequence homology to RNases, the structural components of the exosome are not believed to contribute directly to RNA degradation in vivo. Rather, in yeast, where it has been extensively characterized, 3′ to 5′ exonuclease activity is performed by two exosome-associated RNA exonucleases: Rrp6, which is exclusive to the nucleus, and Dis3, which is present in both the nucleus and the cytoplasm [114,115,116].

The exosome is both structurally and functionally conserved from yeast to humans [117]. Indeed, mutations in the yeast exosomal genes Rrp4, Csl4, or Dis3 can be complemented with the human orthologs [116]. Interestingly, the localization of the exosome exonucleases has diverged over evolutionary history. Rrp6 is present in both the nuclear and cytoplasmic fractions of human cells, and Dis3 has two additional paralogs in humans, Dis3L1 and Dis3L2, which function exclusively in the cytoplasm [118]. Dis3L2, which lacks the exosome-associating PIN domain, operates independently of the larger exosome complex in a separate 3′ to 5′ degradation system which favors terminally uridylated RNAs [119,120,121].

Though the exosome degrades RNAs indiscriminately in vitro, it degrades RNAs in vivo in a regulated fashion by relying on RNA-binding cofactor complexes that bind specific targets and recruit the exosome for degradation [122]. All known exosome cofactor complexes are anchored by helicases which are thought to unwind higher-order RNA structures to permit single-stranded RNA to be inserted into the exosome barrel for decay [123]. Different RNAs are targeted by the exosome in the nucleus, nucleolus, and cytoplasm; therefore, the exosome relies on different cofactors in each subcellular compartment to target these diverse RNAs for decay. Two major complexes, the cytoplasmic Ski and nuclear TRAMP complexes, have been extensively characterized in yeast.

The Superkiller (Ski) complex is the major cytoplasmic exosome cofactor complex in yeast, named for the “superkilling” phenotype of dsRNA viruses, which are lethal to yeast deficient in these genes [124]. The Ski genes were identified before the discovery of the exosome, and some components of the exosome barrel were also assigned Ski names. Though mutants in cofactor Ski genes lead to increased vRNA, this has not yet been definitively linked to exosomal RNA degradation [125]. The Ski complex consists of a DExH/D-box helicase, Ski2, a tetratricopeptide repeat-containing protein, Ski3, and a WD repeat-containing protein, Ski8 [126]. An adaptor G-protein, Ski7, physically links the Ski complex and the exosome and is required for Ski complex-mediated decay [127]. The Ski complex is involved in recruiting the exosome to RNAs targeted for NMD, as well as nonstop decay [128,129,130]. Orthologs for all three Ski genes are present in higher organisms, though their specific targets have not been clearly defined [131,132]. Interestingly, a recent paper found that the human Ski complex-associated helicase SKI2L prevents hyper-activation of RIG-I in uninfected cells, protecting cells from autoimmune activation and patients harboring mutations in this gene presented with anomalously high IFN signatures [133]. Though the exosome was not shown to be required for this activity, it does suggest that the Ski-associated helicase, potentially with the exosome, may serve to protect the intracellular milieu from overactive RIG-I signaling, paralleling the role of the DNA exonuclease TREX, which degrades cytoplasmic DNA to prevent hyper-activation of the DNA sensor cGAS [134,135,136].

The yeast TRAMP (Trf4/5-Air1/2-Mtr4-Polyadenylation) complex, located in the nucleus, has known roles in degrading hypomodified tRNAs, hypoadenylated mRNAs, cryptic unstable transcripts (CUTs), and in the biogenesis of rRNA, snRNA, and snoRNA [72,73,74,122,137,138]. The complex is anchored by a DExD/H box helicase, Mtr4, which binds the other TRAMP components through its arch domain [123,139]. The Zn-knuckle RNA-binding proteins Air1 and Air2 bind specific RNAs and target them for degradation [140]. These two proteins are partially functionally redundant; mutants in each protein accumulate overlapping but non-identical populations of snRNAs, snoRNAs, and mRNAs, and double-mutant strains fail to grow. Trf4 and Trf5 are non-canonical poly(A) polymerases which add 5–6 adenines to RNAs bound to the TRAMP complex [137]. The addition of short poly(A) tails creates an unstructured 3′ end which is thought to facilitate insertion of the RNA into the exosome barrel [141]. This adenylation parallels the role of polyadenylation in *Escherichia coli*, which, unlike eukaryotic polyadenylation, targets RNAs for decay [142,143]. In addition to the canonical TRAMP complex, Mtr4 can form other modular cofactor complexes by associating with the adaptors Nop53 or Utp18, which assist in some rRNA maturation steps [144].

The TRAMP complex is conserved in humans, but nuclear RNA degradation machinery has additional complexity. As in yeast, human TRAMP is composed of a helicase, hMTR4, a zinc-finger Air-like protein, hZCCHC7, and a poly(A) polymerase, hTRF4-1 or hTRF4-2 [145,146]. However, unlike yeast TRAMP, the human TRAMP complex is restricted to the nucleolus, and is only known to process rRNA [145,147]. Most of the yeast TRAMP targets, such as mRNAs, snRNAs, snoRNAs, and promoter upstream transcripts (PROMPTs, which are analogous to yeast CUTs) appear to be regulated in human cells by the nuclear exosome targeting (NEXT) complex, which shares hMTR4 with the TRAMP complex, but also contains the zinc-finger protein hZCCHC8 and the RNA-binding motif protein hRBM7 [145,148,149]. Other targets are likely to exist for mammalian TRAMP-like complexes; murine cells depleted of Mtr4 accumulate adenylated 5′ miRNA fragments, suggesting that adenylation and Mtr4-mediated degradation may be important for these RNAs [150]. The full spectrum of Mtr4-anchored complexes in mammals and the regulation of other classical yeast TRAMP targets (such as misprocessed tRNAs) remain unclear.

## 8. Antiviral Roles for the RNA Exosome and 3′ Decay

Studies have implicated the exosome in antiviral defense. A number of antiviral RNA-binding proteins co-immunoprecipitate with the exosome, suggesting that their mechanism of action may involve exosomal degradation. DDX17 restricts RVFV by binding a miRNA-like stem loop structure encoded in the vRNA [37]. Though its mechanism of restriction is unknown, DDX17 binds to the exosome, suggesting that it directly recruits the exosome to degrade these bound vRNAs [145,151]. DDX60, which is antiviral against vesicular stomatitis virus (VSV), also binds the exosome [39]. However, DDX60 does not depend on the exosome for its antiviral function, but rather bridges vRNA and RLRs to potentiate signaling. The cytidine deaminase AID, which binds the exosome and hepatitis B virus (HBV) RNA in a complex, is antiviral when overexpressed only if the exosome is present, suggesting the possibility that it recruits the exosome to degrade HBV RNA [152]. The zinc-finger antiviral protein (ZAP) binds SINV and retrovirus RNA, as well as components of the exosome [153]. In overexpression systems, ZAP restricts MLV viral replication in an exosome-dependent fashion, as well as affecting the expression and stability of viral luciferase reporters for both MLV and HIV [154,155]. It remains unclear if the exosome is required for the activity of endogenous ZAP or degrades ZAP-bound vRNAs. The cell biology of these factors is largely unexplored, but overexpressed DDX60 and ZAP localize to the cytoplasm, while overexpressed AID binds the exosome in both the nucleus and cytoplasm [39,152,156]. In response to viral infection, DDX17 translocates from the nucleus to the cytoplasm where the vRNAs are located [37].

Recent work has implicated the exosome and components of a canonical cofactor complex in the direct recognition and degradation of specific vRNAs. RNAi screening revealed an antiviral role for exosome core components as well as the TRAMP components Mtr4 and Zcchc7 against three RNA viruses from distinct families, VSV, SINV, and RVFV, in both *Drosophila* and human cells [157]. Though TRAMP components are normally nucleolar, infection with these cytoplasmic viruses leads to the export of hMTR4 and hZCCHC7 to the cytoplasm, where they complex with the exosome and specifically bind viral mRNAs. Further study found that RVFV mRNA is destabilized by the exosome and hZCCHC7, and that the 3′ UTR of RVFV mRNA is sufficient to render a reporter RNA susceptible to exosomal degradation during viral infection. Cell biological studies showed that hZCCHC7 localizes to cytoplasmic punctae during viral infection, suggesting that it may be recruited to RNP granules for its antiviral function.

Taken together, these studies suggest that the exosome is a broad antiviral effector downstream of diverse sensors which bind distinct vRNAs to recruit the exosome for degradation (Figure 1). Though the exosome has been shown to degrade vRNA sensed by some of the proposed exosomal cofactors, such as Mtr4. Zcchc7, and ZAP, much work remains to describe the mechanism and exosomal involvement in antiviral restriction downstream of the other proteins.

## 9. Concluding Remarks

Increasing evidence suggests that the RNA decay machinery plays important roles in antiviral defense. This can involve either direct effects on vRNA stability or indirect regulation of the intracellular milieu. Furthermore, an emerging theme suggests that many RNA binding proteins can be repurposed from their endogenous roles in the nucleus to antiviral roles in the cytoplasm. Future studies are necessary to further elucidate how these RNA binding proteins recognize foreign RNAs and how they interface with the RNA decay machinery to restrict vRNA replication.
